# Management of posterior wall acetabular fractures using the anterior intrapelvic approach with lag screw fixation: A retrospective clinical and radiological study

**DOI:** 10.1097/MD.0000000000045060

**Published:** 2025-10-10

**Authors:** Cagatay Gemci, Emre Gultac, Fatih Ilker Can, Ismail Gokhan Sahin, Cem Yalin Kilinc, Nevres Hurriyet Aydogan

**Affiliations:** a Department of Orthopedics and Traumatology, Faculty of Medicine, Muğla Sitki Koçman University, Muğla, Türkiye.

**Keywords:** acetabular fractures, lag screw, posterior acetabular wall fractures, quadrilateral surface

## Abstract

The management of combined anterior column and posterior wall acetabular fractures requires dual surgical approaches, which may increase operative morbidity. This study evaluated the efficacy and safety of posterior wall lag screw fixation using the anterior intrapelvic (AIP) approach, eliminating the need for a secondary posterior incision. Patients with concomitant anterior column and posterior wall fractures were included in the study by collecting acetabular fractures surgically treated via AIP approach between 2014 and 2019. Posterior wall fractures were subsequently stabilized with lag screws inserted using the same anterior approach, without additional posterior exposure. Patients who had <12 months follow-up data were excluded. The reduction quality was assessed using the Matta radiographic criteria. Functional outcomes were evaluated using the Harris Hip Score and the modified Merle d’Aubigné and Postel scoring system. Postoperative short- and long-term complications were also evaluated. At the 12-month postoperative follow-up, radiographic evaluations confirmed complete fracture union in all patients, without any loss of reduction or signs of posterior instability. According to Matta radiographic criteria, anatomic reduction was achieved in 4 patients (33.3%), good reduction in 6 patients (50%), and poor reduction in 2 patients (16.6%). Functional outcomes showed a mean Harris Hip Score of 87.0 ± 9.3, with 7 patients (58.3%) rated as “excellent,” 2 (16.6%) as “good,” and 3 (25%) as “fair.” The mean modified Merle d’Aubigné and Postel scoring system was 16.41 ± 1.5, with 4 patients (33.3%) graded as “excellent,” 6 (50%) as “good,” and 2 (16.6%) as “fair.” The mean hospital stay was 8.4 ± 4.3 days (range: 5–18), with a mean operative time of 115.8 ± 36 minutes (range: 90–225) and mean intraoperative blood loss of 580 ± 555 mL (range: 250–2300). The AIP approach with posterior wall lag screw fixation provides a viable solution for complex acetabular fractures, delivering favorable clinical and radiological results while avoiding posterior incisions. Preoperative planning and precise screw placement are critical for success. Further studies are recommended to refine patient selection and improve surgical protocols.

## 1. Introduction

Acetabular fractures, particularly those involving the posterior column, present significant challenges in orthopedic trauma surgery due to their complex anatomy and the necessity for precise reduction and stable fixation to restore hip joint function. The anterior intrapelvic (AIP) approach, also known as the modified Stoppa approach, has gained prominence for accessing and managing such fractures. This approach allows for direct visualization and manipulation of the quadrilateral surface and posterior column, thereby facilitating accurate fracture reduction.^[1]^ Additionally, the AIP approach has been utilized for the reduction and fixation of displaced acetabular fractures, providing adequate exposure to address both anterior and posterior injuries. This approach facilitates the use of various fixation techniques including lag screws and plates to achieve stable osteosynthesis.^[1–3]^

In complex acetabular fractures, combinations of both anterior and posterior columns and wall fractures can be seen. In the presence of posterior wall fractures accompanying anterior column and wall fractures, posterior wall reduction and fixation are usually applied in addition to anterior incisions by adding the posterior Kocher Langenbeck (K-L) approach.^[4,5]^ Due to the risk of additional potential complications, such as sciatic nerve damage, heterotopic ossification, bleeding, infection, and abductor mechanism damage due to the extra posterior incision, we hypothesized that it would be advantageous to fix these types of fractures with only the AIP approach and fix the posterior wall fracture with a lag screw from anterior to posterior in addition to the fixation of the anterior structures.

Lag screw fixation targeting the posterior wall via the quadrilateral surface is a technique employed to achieve stable fixation in non-displaced, non-fragmented, indirectly reducible acetabular fractures. This method involves inserting screws from the inner table of the pelvis, traversing the quadrilateral surface, and anchoring them to the posterior wall. Such fixation aims to provide stability, while minimizing additional soft tissue disruption. Studies have demonstrated that medial surface plating of the posterior wall through the AIP approach offers good stability and reduces the morbidity associated with acetabular fractures.^[6]^

Preoperative planning is crucial for successful placement of posterior wall screws via an anterior approach. Assessing the fracture pattern and determining the appropriate screw trajectory are essential to ensure effective fixation and avoid complications. Research indicates that careful preoperative assessment enhances the eligibility of fractures using this fixation method.^[7]^

In summary, the AIP approach combined with lag screw fixation on the quadrilateral surface, targeting the posterior wall, is a viable surgical strategy. This technique offers the advantages of avoiding complications associated with the posterior approach, shorter operation duration, and potentially reduced surgical morbidity.

To the best of our knowledge, the specific approach of posterior wall lag screw fixation solely via the AIP route has not been previously described in the literature. We hypothesized that this novel approach could achieve stable fixation and good outcomes while avoiding the morbidity associated with additional posterior exposure. In this study, we aimed to examine the advantages, disadvantages, complications, and radiological and functional results of the quadrilateral plate placement lag screw technique, which we applied to complex acetabular fractures that can be reduced with indirect maneuvers or accompanied by non-displaced posterior wall fractures.

## 2. Materials and methods

This study was approved by the institutional ethics committee and was conducted in accordance with the principles of the Declaration of Helsinki. The medical records of all patients who underwent acetabular fracture surgery between January 2014 and March 2019 were retrospectively reviewed. Medical records of 152 patients with acetabular fractures were collected from our institution’s database. Fractures were classified using the Letournel Classification system.^[8]^ Isolated posterior wall and/or column fractures, isolated anterior wall and/or column fractures, transverse, T-type, and both-column fractures without posterior wall fracture, pathological fractures, pediatric patients, patients aged > 65 years, patients with incomplete medical records, and patients who did not regularly attend checkups were excluded from the study. Posterior wall fractures in the remaining patients were examined, and those with multiple fragments and those who could not be reduced indirectly and required open posterior reduction and plating were also excluded from the study. After the exclusion criteria, patients treated with an anterior pelvic approach and posterior wall fracture fixation using lag screws, with a minimum follow-up of 12 months, were included. Reduction quality was assessed using Matta Reduction Criteria^[9]^ and functional outcomes were evaluated using the Harris Hip Score (HHS) System and the modified Merle d’Aubigné and Postel scoring system (MDPS).^[10]^

Preoperative and postoperative imaging included pelvic anteroposterior (AP), inlet, outlet, obturator oblique, and iliac oblique radiographs, and computed tomography scans. Additional injuries were identified in 6 patients (50%), including fractures of the distal radius, calcaneus, vertebrae, and sacrum, and vascular injuries. Sciatic or peroneal nerve damage was not observed.

### 2.1. Surgical procedure

All patients were operated on by the same surgeon within 2 weeks of injury (mean interval: 5.25 ± 3.4 days). Standard preoperative antibiotic prophylaxis with 1 g of intravenous cefazolin was administered and continued postoperatively for 10 days. Deep vein thrombosis (DVT) prophylaxis with enoxaparin was administered until ambulation resumed. The AIP approach was used in all cases. Posterior wall fractures were stabilized using lag screws after determining safe screw placement through preoperative pelvic modeling using the Autodesk Fusion 360 software(Autodesk, Inc., San Francisco).

**Figure 1.**
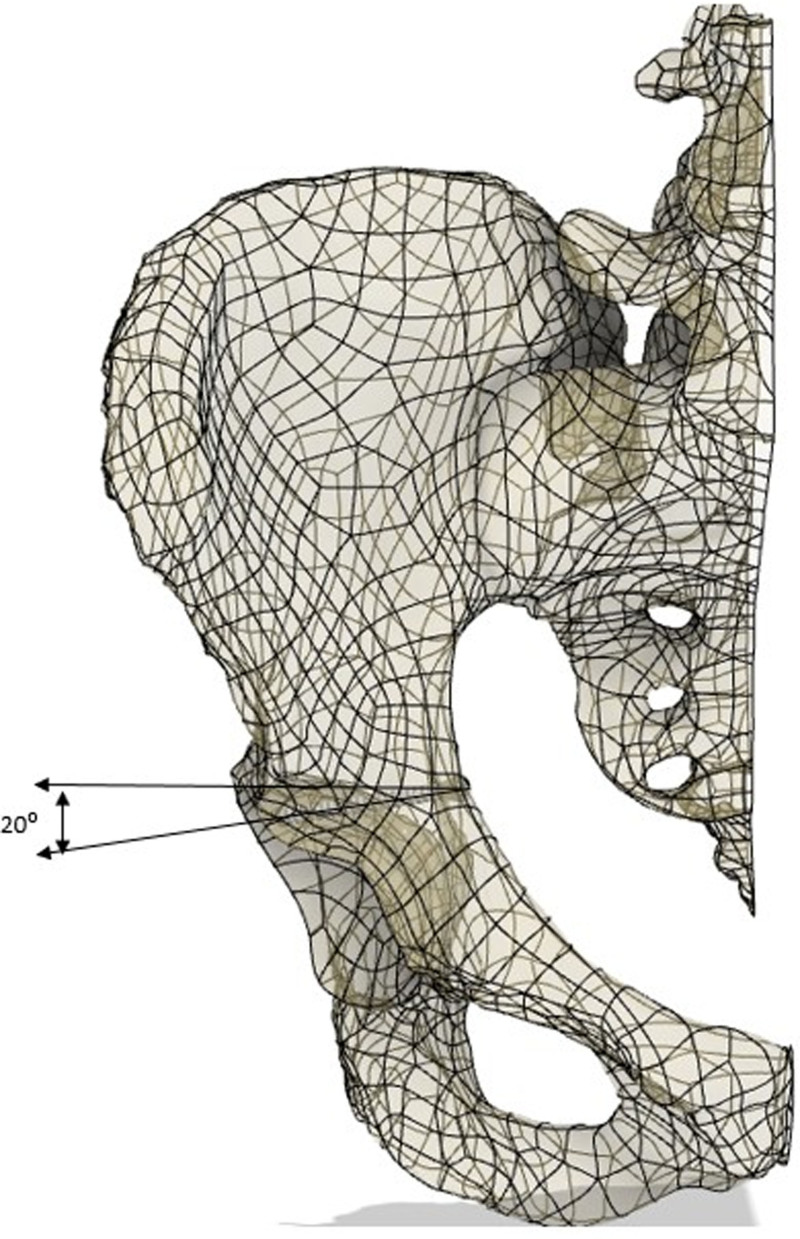
The entry point of the posterior wall lag screw.

**Figure 2.**
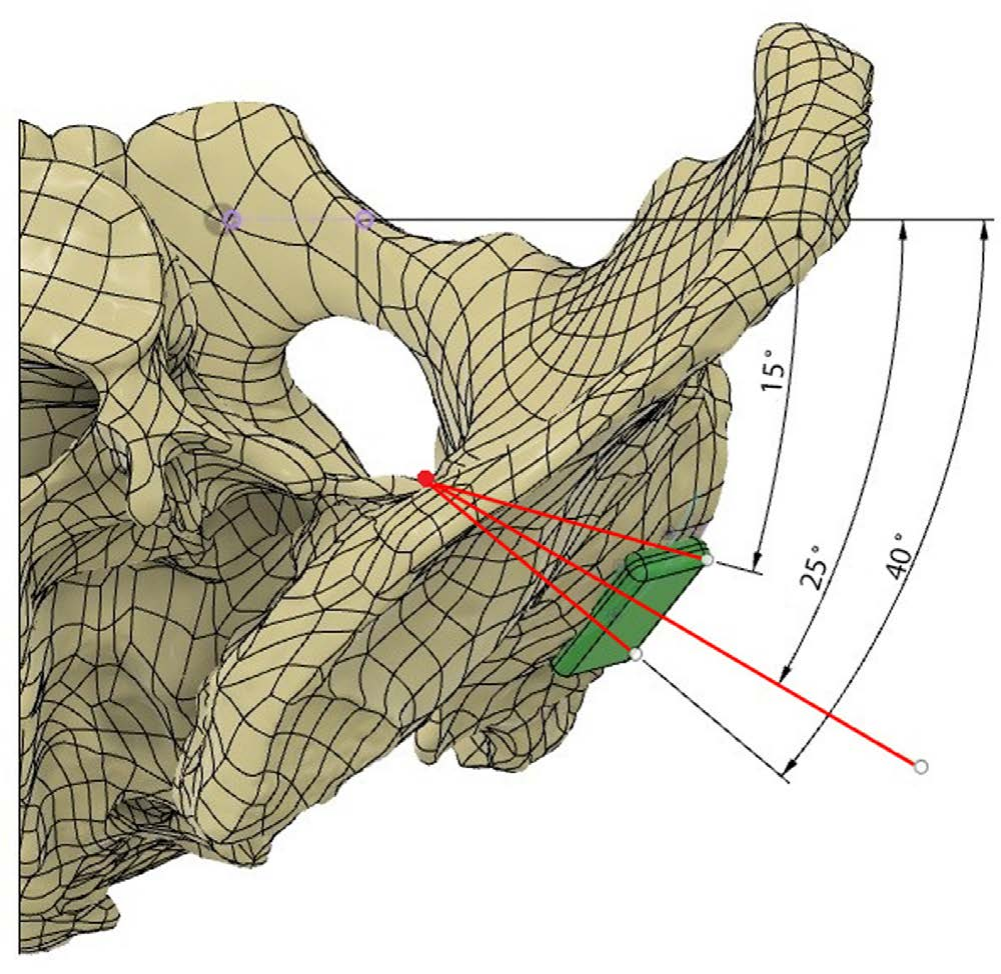
The direction of the posterior wall lag screw and the safe zone for screwing (sagittal plane).

**Figure 3.**
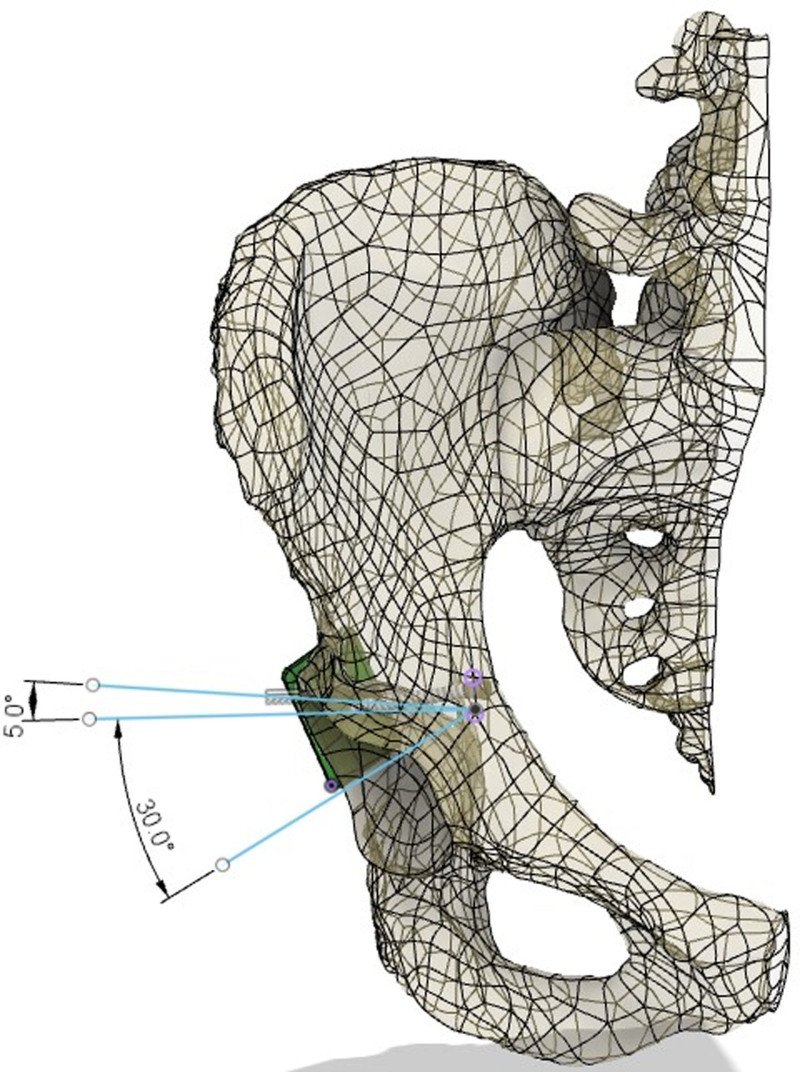
The direction of the posterior wall lag screw and the safe zone for screwing (horizontal plane).

**Figure 4.**
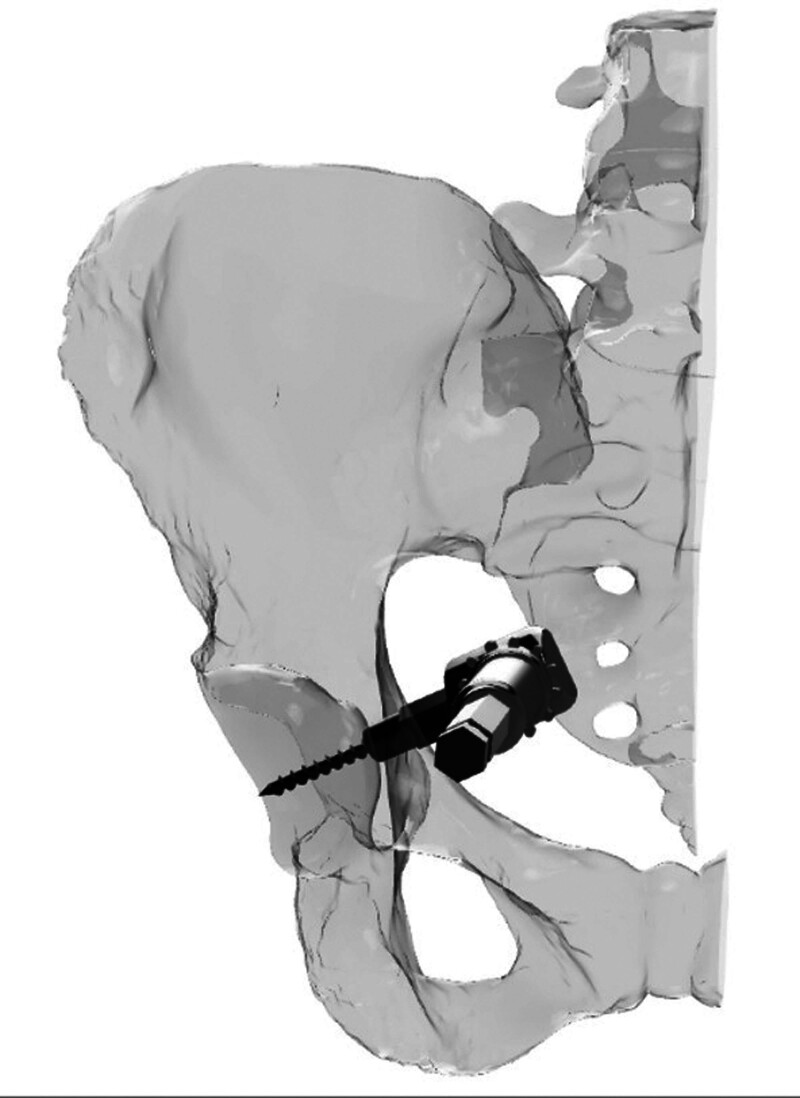
Drilling of the posterior wall using a 105° drill bit via anterior intrapelvic approach.

**Figure 5.**
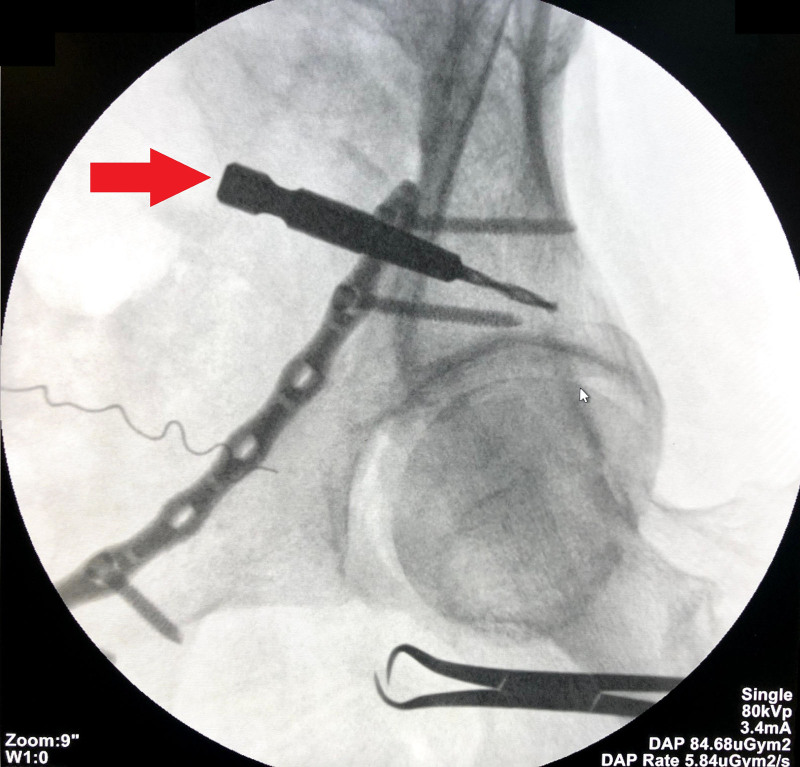
Iliac oblique fluoroscopy image showing the drill bit left in the screw entry point (red arrow).

**Figure 6.**
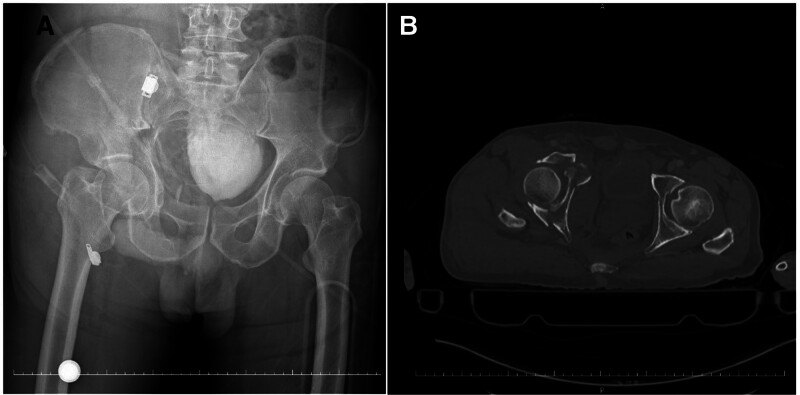
(A) Preoperative anteroposterior pelvic X-ray image. (B) Preoperative axial computed tomography image.

**Figure 7.**
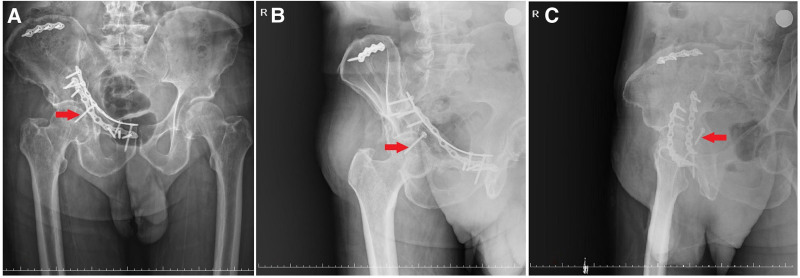
(A) Postoperative anteroposterior pelvic X-ray image. (B) Postoperative obturator oblique pelvic X-ray image. (C) Postoperative iliac oblique pelvic X-ray image (red arrows: posterior wall lag screw).

During modeling, a safe rectangular area was created starting 1 mm medial to the posterior wall border of the acetabulum. The entry point on the quadrilateral surface was determined approximately 1.5 to 2.5 cm inferior to the point on the pelvic ring of the line drawn parallel to the ground from the roof of the acetabulum in the pelvis AP image (Fig. 1). Then, the posterior wall was targeted in a way that would prevent penetration into the hip joint and the direction of the screw was calculated using the pelvic modeling system. From this point, it was determined that the drill bit should be directed 25 (15–40) degrees anteriorly in the sagittal plane and approximately 20 (5–30) degrees caudally in the horizontal plane (Figs. 2 and 3). Owing to orientation difficulties caused by the narrowness of the operation area, a 105° angled drill bit was used instead of a conventional drill (Fig. 4).^[11]^ All patients underwent surgery under general anesthesia in the supine position. After retracting the bladder by applying an AIP vertical incision, the corona mortis was dissected and ligated. The iliopectineal fascia was dissected, the obturator bundle protected, and the quadrilateral surface visualized. The fractures on the quadrilateral surface were reduced and suprapectineal, and if necessary, infrapectineal plates were placed. If the fracture on the posterior wall was displaced, a 3 cm longitudinal mini-incision was made approximately 3 cm cranial to the greater trochanter and the posterior acetabulum was reached via blunt dissection. Subsequently, reduction was achieved using a ball-spike pusher. If the fracture was nondisplaced, to prevent loss of concentric reduction in the fracture line that may occur during drilling and screwing, the screw was achieved by applying pressure to the fracture fragment on the posterior wall with a ball spike pusher. Pelvic AP, obturator oblique, and iliac oblique radiographs were obtained with fluoroscopy, leaving the drill bit inside the pelvis (Fig. 5). The entry point was evaluated on the AP image, orientation of the drill bit to the posterior wall was assessed on the obturator oblique image, and possible joint penetration was assessed on the iliac oblique radiograph. After confirming that the drilling direction was appropriate, a 3.5 mm compression screw with a washer was placed. After the screw was applied, the adequacy of screw placement was checked using fluoroscopy images (Fig. 6). The placement of the compression screw can be observed on X-rays in Figure 7.

Clinical outcomes were assessed using the HHS and MDPS at routine clinical follow-ups. The aforementioned data for the 12. Monthly follow-ups were collected from the patients’ medical records.

No baseline functional assessments were performed before surgery; thus, postoperative scores represent the patients’ status at follow-up. A control group treated with the conventional Kocher-Langenbeck approach was not available for this study, which is acknowledged as a limitation.

### 2.2. Statistical analysis

Statistical analysis was performed using the IBM SPSS version 22.0 software (IBM Corp., Armonk). Descriptive data were presented in mean ± standard deviation, median (min–max) or number and frequency, where appropriate. Due to the novelty of our surgical technique, the study has a small sample size, therefore no formal power analysis was performed.

## 3. Results

The study included 12 patients with injuries classified according to the Judet–Letournel system. Five patients (41.6%) had posterior wall fractures with associated both-column fractures, 3 (25%) had transverse + posterior wall fractures, 2 (16.6%) had T-type fractures, and 2 (16.6%) had anterior column-posterior hemitransverse fractures. Fractures were located on the right side in 8 patients (66.6%) and the left side in 4 patients (33.3%).

The cohort consisted of 1 female (8.3%) and 11 males (91.6%), with a mean age of 51.4 ± 7.7 years (range: 39–62). The mechanisms of injury included falls from heights (33%), motor vehicle accidents (33.3%), crush injuries (16.6%), farm animal-related injuries (8.3%), and falls from stairs (8.3%).

The mean hospital stay was 8.4 ± 4.3 days (range: 5–18). The mean operation duration was 115.8 ± 36 minutes (range: 90–225), and the mean intraoperative blood loss was 580 ± 555 mL (range: 250–2300).

According to the postoperative 12. Follow-up radiological evaluations confirmed fracture healing in all the patients, with no evidence of reduction or posterior instability.

**Figure 8.**
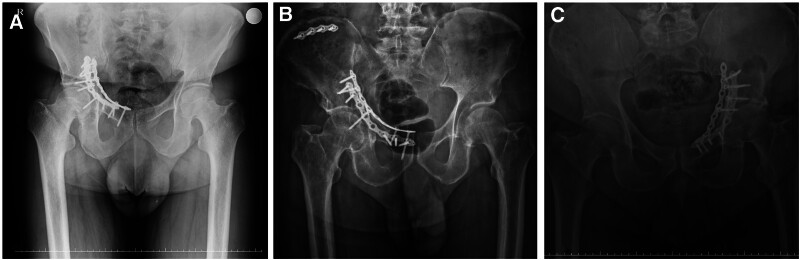
(A) X-ray image presenting the anatomic result of a patient at postoperative 12-month. (B) X-ray image presenting the good result of a patient at postoperative 12-month. (C) X-ray image presenting the poor result of a patient at postoperative 12-month.

Radiological evaluations showed anatomic reduction in 4 patients (33.3%), good reduction in 6 patients (50%), and poor reduction in 2 patients (16.6%) based on Matta reduction criteria. The mean HHS was 87.0 ± 9.3, with 7 patients (58.3%) scoring “excellent,” 2 (16.6%) “good,” and 3 (25%) “fair.” The MDPS averaged 16.41 ± 1.5, indicating “excellent” results in 4 patients (33.3%), “good” in 6 (50%), and “fair” in 2 (16.6%; Table 1). A representative X-ray image presenting the anatomic, good and poor results of patients at postoperative 12- month can be seen in Figure 8A–C, respectively.

**Table 1:** Demographic data, fracture characteristics, hospital stay, operation duration and surgical outcomes.

Parameter	Patient counts and results
Fracture classification (Judet–Letournel)	
Posterior wall + both-column fractures	5 (41.6%)
Transverse + posterior wall fractures	3 (25%)
T-type fractures	2 (16.6%)
Anterior column-posterior hemitransverse fractures	2 (16.6%)
Fracture location	
Right side	8 (66.6%)
Left side	4 (33.3%)
Gender distribution	
Male	11 (91.6%)
Female	1 (8.3%)
Mean age	51.4 ± 7.7 yr (range: 39–62)
Mechanism of injury	
Falls from height	4 (33.3%)
Motor vehicle accidents	4 (33.3%)
Crush injuries	2 (16.6%)
Farm animal-related injuries	1 (8.3%)
Falls from stairs	1 (8.3%)
Hospital stay	Mean: 8.4 ± 4.3 d (range: 5–18)
Operation duration	Mean: 115.8 ± 36 min. (range: 90–225)
Intraoperative blood loss	Mean: 580 ± 555 mL (range: 250–2300)
Radiological outcomes (Matta criteria)	
Anatomic reduction	4 (33.3%)
Good reduction	6 (50%)
Poor reduction	2 (16.6%)
Functional outcomes	
Harris Hip Score (HHS)	Mean: 87.0 ± 9.3
Excellent	7 (58.3%)
Good	2 (16.6%)
Fair	3 (25%)
Modified Merle D’Aubigné and Postel Score	Mean: 16.41 ± 1.5
Excellent	4 (33.3%)
Good	6 (50%)
Fair	2 (16.6%)

Postoperative complications included no instances of heterotopic ossification, DVT, infection, or nerve injury.

## 4. Discussion

This study assessed the clinical and radiological outcomes of posterior wall acetabular fracture fixation via the AIP approach, using a lag screw on the quadrilateral surface through the posterior acetabular wall. The findings demonstrated a high rate of anatomic and good fracture reduction, consistent with the prior literature. This discussion contextualizes these results in relation to existing studies, highlighting the similarities, differences, and implications for clinical practice.

Our study achieved anatomic reduction in 33.3% of patients and good reduction of 50% based on Matta criteria. Similar outcomes were reported by Hazra et al, who found that medial surface plating of the posterior column via the AIP approach resulted in excellent reduction quality.^[6]^ Their study highlighted the advantages of direct visualization, facilitating accurate alignment, and stable fixation. Trikha et al also found that early operative treatment using this approach achieved good to excellent reduction in 90.62% of patients.^[12]^

The average HHS in this study was 87.0 ± 9.3, with 58.3% achieving “excellent” scores. Comparable functional outcomes were documented by Can et al, in which bilateral acetabular fractures treated using the modified Stoppa approach yielded satisfactory long-term hip scores.^[1]^ These results underscore the effectiveness of the AIP approach for restoring hip joint function after complex acetabular fractures. This finding aligns with the results reported by Sagi et al, who documented 91% good-to-excellent clinical outcomes.^[13]^

The modified Stoppa approach employed consistently in this study is supported by Guo et al as a reliable method for addressing complex acetabular fractures.^[2]^ Their research confirmed that this technique minimizes iatrogenic soft-tissue damage while providing broad surgical exposure. Additionally, Krappinger et al emphasized that posterior column screw placement via an anterior approach allows for secure fixation with reduced neurovascular risks.^[7]^ Gras et al further validated the use of pre-shaped suprapectineal plates to improve the reduction quality.^[14]^

Preoperative computed tomography and 3-dimensional modeling significantly contributed to safe screw placement, echoing the findings of Krappinger et al.^[7]^ Their research demonstrated that virtual fracture reconstructions enhance the precision of surgical planning, potentially reducing the operative time and improving fixation accuracy.

All the patients in our study achieved fracture healing without loss of reduction or posterior instability at the latest follow-up. Comparable long-term radiological stability has been documented in studies focusing on the application of the AIP approach. Hazra et al reported sustained radiological integrity and minimal post-traumatic arthritic changes, suggesting that precise reduction and stable fixation mitigate long-term complications.^[6]^

The mean operative time in our study was 115.8 ± 36 minutes, with an average blood loss of 580 ± 555 mL. These parameters are consistent with the findings of previous studies, which also reported similar operative durations and intraoperative hemorrhage levels during acetabular fixation procedures.^[1,15]^

The findings of this study reinforce the AIP approach combined with lag screw fixation as a viable strategy for the management of complex acetabular fractures. Key considerations in clinical practice include meticulous preoperative planning, advanced imaging for safe screw placement, and adherence to established surgical protocols to minimize complications. In complex acetabular fractures, a combination of anterior and posterior column and wall fractures frequently coexist, necessitating dual approaches for adequate management. Traditionally, posterior wall fractures accompanying the anterior column and wall fractures are treated with additional posterior incisions, such as the K-L approach, to achieve proper reduction and fixation. However, this technique presents risks, including sciatic nerve damage, heterotopic ossification, increased blood loss, infection, and potential damage to the abductor mechanism. Given these risks, it has been hypothesized that using only the AIP approach for fixation could be advantageous, especially when coupled with lag screw fixation from the anterior to the posterior for posterior wall fractures. This strategy minimizes the need for extensive posterior exposure, while providing sufficient access to address complex fracture patterns.

Additionally, Kistler and Sagi reported effective posterior column reduction using specialized reduction clamps and fixation devices through the anterior approach, eliminating the need for posterior exposure in select cases.^[16]^

Based on the relevant literature, the K-L approach combined with the AIP approach has been associated with notable complication rates, including surgical site infections, heterotopic ossification, nerve injuries, blood loss, and prolonged surgery. Avilucea et al reported that up to 8.8% of patients developed infections when treated with the K-L approach, with both superficial and deep infections reported.^[17]^ Paksoy et al reported heterotopic ossification rates of as 14% to 50% in patients undergoing K-L approaches alone.^[18]^ In a study by Alexa et al, sciatic nerve injuries occurred in up to 7% of the patients, which is a significant risk factor when performing the K-L approach.^[19]^ Additionally, greater blood loss and prolonged surgery are other issues associated with combined anterior and posterior acetabular approaches. Using dual approaches significantly increased operative time and blood loss due to extensive dissection, according to a study by Salameh et al.^[20]^

In the current study, no major complications such as DVT, infections, or nerve injuries were observed. This finding aligns with the outcomes reported by Soni et al, who reviewed modified Stoppa approach applications and noted low complication rates attributed to reduced surgical trauma and improved visualization.^[3]^ Similarly, Chen et al reported minimal postoperative complications in geriatric patients using a combined anterior-intrapelvic approach.^[21]^

Our findings align with these results, supporting the notion that anterior-only approaches reduce surgical morbidity, while maintaining high rates of fracture reduction and stability. Future research should further explore this approach through multicenter trials and longer follow-up periods to refine the patient selection criteria and optimize surgical protocols.

Although our study provides valuable insights, its retrospective single-center design, small sample size, lack of baseline functional data and absence of a control group limit the generalizability of the results. Future studies should explore larger multicenter cohorts and conduct randomized controlled trials to establish definitive treatment protocols. Additionally, longer follow-up durations would help clarify the impact of surgical techniques on the progression of posttraumatic osteoarthritis.

## 5. Conclusion

The AIP approach with posterior wall lag screw fixation offers a promising surgical technique for complex acetabular fractures, delivering high rates of anatomic and good reductions, while minimizing surgical morbidity. Preoperative 3-dimensional modeling and careful surgical planning are critical for safe and effective screw placement. This method avoids the need for posterior incisions and reduces associated complications such as nerve damage, excessive blood loss, and postoperative infections. Future multicenter studies with larger sample sizes and longer follow-up durations are recommended to validate these findings and optimize the surgical protocols.

## Author contributions

**Conceptualization:** Cagatay Gemci, Cem Yalin Kilinc.

**Data curation:** Emre Gultac, Fatih Ilker Can.

**Formal analysis:** Emre Gultac, Cem Yalin Kilinc.

**Investigation:** Cem Yalin Kilinc.

**Methodology:** Cagatay Gemci, Fatih Ilker Can, Ismail Gokhan Sahin.

**Software:** Fatih Ilker Can, Ismail Gokhan Sahin.

**Supervision:** Cem Yalin Kilinc.

**Writing – original draft:** Cagatay Gemci, Emre Gultac.

**Writing – review & editing:** Nevres Hurriyet Aydogan.
